# Autonomic imbalance assessed by time-domain heart rate variability indices in primary Raynaud’s phenomenon

**DOI:** 10.5830/CVJA-2015-032

**Published:** 2015

**Authors:** Karabacak Kubilay, Erkan Kaya, Murat Kadan, Gokhan Arslan, Ufuk Demirkilic, Murat Celik

**Affiliations:** Department of Cardiovascular Surgery, Gulhane Military Academy of Medicine, Ankara, Turkey; Department of Cardiovascular Surgery, Gulhane Military Academy of Medicine, Ankara, Turkey; Department of Cardiovascular Surgery, Gulhane Military Academy of Medicine, Ankara, Turkey; Department of Cardiovascular Surgery, Gulhane Military Academy of Medicine, Ankara, Turkey; Department of Cardiovascular Surgery, Gulhane Military Academy of Medicine, Ankara, Turkey; Department of Cardiology, Gulhane Military Academy of Medicine, Ankara, Turkey

**Keywords:** heart rate variability, autonomic nervous system, Raynaud’s phenomenon

## Abstract

**Objectives:**

The pathogenesis of primary Raynaud’s phenomenon (RP) seems to be multifactorial and autonomic nervous dysfunction is one factor. Heart rate variability (HRV) is one of the most reliable parameters to demonstrate autonomic dysfunction. Our aim was to evaluate the time-domain HRV in patients with primary RP.

**Methods:**

A time analysis of HRV was performed in patients with primary RP and age- and gender-matched healthy controls. The results of the study and control group were compared.

**Results:**

Thirty patients with primary RP [all men, median (IQR) age: 21 (2) years) and 31 age- and gender-matched healthy controls (median (IQR): 21(3) years) were enrolled in the study. We found a statistically significant difference between the primary RP patients and control subjects in terms of time-domain HRV parameters (*p* < 0.05 for all).

**Conclusion:**

Our study showed the presence of autonomic nervous dysfunction of heart function in patients with primary RP.

## Objectives

Raynaud’s phenomenon (RP) is a vascular disorder depicted by a repeated course of fading of the fingers and/or toes, which is caused by reversible vasospasm.^1^ The prevalence of RP varies between three and 4%.^2^ Primary RP (Raynaud’s disease) occurs as an isolated symptom, whereas secondary RP (Raynaud’s syndrome) is associated with another disease or condition. About 8–9% of all RP are primary cases.^2^,^3^ Primary RP has a greater female predominance and is seen at an earlier age than secondary RP.^2^

The pathogenesis of RP is not fully elucidated. Intravascular and neural mechanisms play a pivotal role in this process.^4^ Increased sympathetic activity is often thought to be one of the causes in the aetiology of primary RP. Increased sympathetic activation in the chronic phase may also lead to desensitisation of the sinus node to neural input and parasympathetic tone, thereby causing autonomic dysfunction.

Heart rate variability (HRV) analysis shows sympathovagal balance and has been used as an easy, non-invasive and reliable test for the identification of dysfunction of the autonomic nervous system in certain diseases. Since sympathovagal balance is affected in favour of sympathetic activation in patients with primary RP, in this study, we evaluated the baseline function of the autonomic nervous system assessed by 24-hour HRV analysis in patients with primary RP.

## Methods

Patients referred to our Cardiovascular Department for suspicion of primary RP during the period October 2012 to May 2013 were evaluated in this study. The evaluation of past medical history, physical examination, an initial 12-lead ECG and then 24-hour Holter monitoring for HRV analysis were performed in all patients.

Diagnosis of Raynaud’s phenomenon was confirmed with the three-phase cold test. All patients were also evaluated for secondary RP. On physical examination, all patients were examined for skin ulceration, telangiectasia, muscle weakness and connective tissue diseases such as scleroderma.

In our study, we tried to exclude metabolic factors that may affect the heart rate. Patients with hypothermic or hyperthermic status, injury, anaemia, infection or history of any chronic disease, such as connective tissue disorder or diabetes mellitus, were excluded. Also, structural heart disease, supraventricular or ventricular arrhythmias, atrial fibrillation, sick sinus syndrome, atrioventricular block, haematological or neurological disease, the use of any drug that has an impact on heart rate (such as beta-adrenergic blockers and anti-arrhythmic drugs) were accepted as other exclusion criteria.

All HRV measurements were performed at rest. The patients were advised not to take part in vigorous exercise during the HRV measurement.

The control group consisted of age- and gender-matched healthy subjects. An informed consent was obtained from all subject enrolled in this study, which was conducted in accordance with the regulations of Declaration of Helsinki. The regional ethics committee of our Institute approved the study protocol.

## HRV analysis

A 24-hour Holter ambulatory ECG monitoring (Rozinn RZ 152 digital Holter recorder, Rozinn Electronics, Inc, Glendale, NY, USA) with a sampling rate of 1 024 Hz was performed on all subjects enrolled in the study. The HRV was automatically analysed with software of the same device.

Standard deviation of all R–R intervals (SDNN), standard deviation of the successive N–N differences (SDSD), standard deviation of the averages of the R–R intervals in all five-minute segments of R–R intervals (SDANN), the mean of all the five-minute standard deviations of N–N (normal R–R) intervals during the 24-hour period (SDNN index), the mean square root of the difference of successive R–R intervals (RMSSD), successive N–N intervals differing more than 50 ms (NN50 count), and the proportion of adjacent normal R–R intervals < 50 ms (pNN50) were defined as the time-domain variables of HRV analysis. HRV analyses of the patients with primary RP were compared with those of healthy controls.

## Statistical analysis

The data were tested for normal distributions using the Kolmogorov–Smirnov test. The continuous variables are presented as means ± standard deviation (SD) and the categorical variables as percentages. The chi-square test was used to compare categorical data and the independent-samples *t*-test was used to compare quantitative data. Spearman’s and Pearson’s correlation coefficients were used to perform univariate correlation. A *p*-value < 0.05 was considered statistically significant. All statistical analyses and calculations were performed using the Statistical Package for Social Sciences version 20.0 (SPSS, Chicago, Illinois, USA).

## Results

Thirty patients (median age 21 years, IQR: 2) in whom the three-phase cold test was positive, were diagnosed as primary RP and enrolled in the study. The average duration of primary RP patients’ symptoms was 3 (2.13) years [median (IQR)]. The control group consisted of 31 healthy subjects (median age 21 years, IQR: 3). There was no statistically significant difference between primary RP patients and healthy control subjects regarding basal demographic characteristics (Table 1).

**Table 1:** Basal demographic and clinical characteristics of the two groups

	Primary RP (n = 30)	Control (n = 31)	p-value
Age (years), median (IQR)*	21 (2)	21 (3)	0.381
Male, n (%)	30 (100)	31 (100)	NA
SBP (mmHg)	116.76 ± 10.45	119.41 ± 10.13	0.318
DBP (mmHg)	74.43 ± 7.81	75.54 ± 9.30	0.615
Smoking, n (%)	10 (33.3)	7 (22.6)	0.258
Duration of primary RP (years), median (IQR)*	3 (2.13)	-	NA

*Data without normal distribution were expressed as median (interquartile range).

RP: Reynaud’s phenomenon, SBP: systolic blood pressure, DBP: diastolic blood pressure IQR: interquartile range.

All participants were in sinus rhythm without any arrhythmias. Statistical analysis of the HRV analyses showed a significant decrease in time-domain variables of SDNN, RMSDD, NN50 count, PNN50 and SDNN index between the two groups (*p* < 0.05 for all) (Table 2).

**Table 2:** Comparison of time-domain HRV indices between the two groups

	Primary RP (n = 30)	Control (n = 31)	p-value
Mean heart rate (bpm)	76.80 ± 12.99	76.22 ± 11.74	0.857
SDNN (ms)	145.09 ± 40.65	177.55 ± 66.18	0.025
SDSD (ms)	46.43 ± 24.03	63.40 ± 42.04	0.059
NN50 count (%)	11085.53 ± 10246.89	19302.74 ± 12717.30	0.007
RMSDD (ms)	42.58 ± 22.76	63.39 ± 42.04	0.020
SDANN (ms)	93.33 ± 65.78	79.06 ± 75.89	0.437
SDNN index	55.71 ± 24.50	82.03 ± 34.25	0.001
pNN50	13.30 ± 12.74	21.92 ± 16.86	0.028

SDNN: standard deviation of all R–R intervals, SDSD: standard deviation of the successive N–N differences, NN50 count: successive N–N intervals differing more than 50 ms, RMSSD: the mean square root of the difference of successive R–R intervals, SDANN: standard deviation of the averages of the R–R intervals in all five-minute segments of R–R intervals, SDNN index: the mean of all the five-minute standard deviations of N–N (normal R–R) intervals during the 24-hour period, pNN50: the proportion of adjacent normal R–R intervals < 50 ms.

Univariate correlation analysis showed that the presence of primary RP was moderately correlated with SDNN (*r* = 0.287, *p* = 0.025), RMSDD (*r* = 0.297, *p* = 0.020), NN50 count (*r* = 0.340, *p* = 0.007), PNN50 count (*r* = 0.281, *p* = 0.028) and SDNN index (*r* = 0.409, *p* = 0.001). We did not find any significant correlation between the duration of Raynaud’s phenomenon and the timedomain HRV indices (*p* > 0.05 for all).

To demonstrate the independent effect of time-domain HRV indices on the presence of primary RP, we performed a multivariate logistic regression analysis using the LR method, based on independent variables likely to affect the level of mean platelet volume. In multivariate analysis, SDNN index (β = 1.138, 95.0% CI = 1.049–1.235, p = 0.002) and PNN50 (β = 0.881, 95.0% CI = 0.785–0.989, *p* = 0.032) were the only independent variables (Table 3).

**Table 3:** Univariate analysis and multivariate logistic regression analysis based on independent variables likely to affect the presence of primary RP

	*Univariate analysis*		Multivariate analysis*		
*Variable*	*r*	*p*	*OR*	*95% CI*	*p*
SDNN (ms)	0.287	0.025	0.979	0.955–1.004	0.107
RMSDD (ms)	0.297	0.020	1.012	0.957–1.069	0.684
SDNN index	0.409	0.001	1.138	1.049–1.235	0.002
NN50 count (%)	0.340	0.007	1.000	1.000–1.000	0.711
pNN50	0.281	0.028	0.881	0.785–0.989	0.032

square root of the difference of successive R–R intervals, SDNN index: the mean of all five-minute standard deviations of N–N (normal R–R) intervals during the 24-hour period, NN50 count: successive N–N intervals differing more than 50 ms, pNN50: the proportion of adjacent normal R–R intervals < 50 ms. *p-value at the last step, where the independent variables remained in the backward LR multivariate regression model.

## Discussion

The pathogenesis of primary RP appears to be multifactorial. The endothelium, smooth muscle, circulating mediators, and autonomic and sensory nerves play a pivotal role in maintaining vasomotor homeostasis. Disturbance in these factors may lead to vasospasm of the small arteries and arterioles and to the manifestation of RP.

Since Maurice Raynaud, a French physician, first described Raynaud’s phenomenon in 1862, sympathetic nervous system over-reactivity has been suggested as the most common cause of the disease. Besides sympathetic up-regulation, impaired parasympathetic activation has also been blamed.^5^ Therefore, it is suggested that the autonomic nervous system seems to have a pivotal role in the pathogenesis of Raynaud’s phenomenon.^6^-^8^ However, it should be noted that the autonomic nervous system may not be affected to the same degree in all patients with RP.

The cyclic changes in the sinus node rate over time are defined as heart rate variability. HRV analysis provides information on the balance between sympathetic and parasympathetic innervation of the heart and has been extensively used as an indirect method for the determination of cardiac autonomic function.^9^ Physical and mental stress, exercise, and respiratory and metabolic changes are associated with autonomic tone of heart rate.^10^,^11^

To define the role of the autonomic nervous system in the pathogenesis of certain diseases, a number of studies have used HRV analysis. However, the results may not agree, presumably due to methodological differences. HRV analysis can be carried out by two different methods, time-domain and frequencydomain methods. The frequency-domain method separates heart rate signals by frequency and density. Although its basic principle is simple, it is technically difficult and complex.

The time-domain method is based on analysis of the interval between normal pulses in 24-hour ECG recording. Low-frequency (LF) HRV is an index of sympathetic activity, whereas high-frequency (HF) HRV reflects parasympathetic activity. Increased LF:HF ratio is characterised by an autonomic nervous dysfunction.^12^

Among time-domain HRV indices, SDNN, SDANN and SDNN index reflect the heart rate, and their decrease is related to diminished vagal and increased sympathetic modulation of the sinus node.^13^ In this study, we used the time-domain method and found that time-domain HRV variables differed significantly, showing increased sympathetic activity in patients with primary RP.

Among these variables, intervals during the 24-hour period (SDNN index) and the proportion of adjacent normal R–R intervals < 50 ms (pNN50) were found to be independent variables in multivariate logistic regression analysis. However, it is obvious that the results of the study will be elucidated more after including frequency-domain HRV indices. Despite this limitation, our study gives inspiration for further larger sample sized prospective studies.

Assessment of the autonomic nervous system may play an important role in understanding the underlying mechanism of primary RP. Primary RP patients may have higher resting sympathetic tone or decreased parasympathetic tone. Autonomic parameters of the cardiovascular system can be non-invasively assessed with HRV. The clinical course and prognosis of various cardiac and systemic disorders could be obtained with this assessment.

Many articles in the literature have speculated that HRV could be used in various cardiac and non-cardiac diseases for autonomic regulation, but a limited number of studies have used HRV on patients with primary RF. Koszewicz *et al*. found that patients with primary RP did not have the autonomic stability found in healthy individuals.^3^ In another study, Ferri *et al*.^14^ studied HRV changes in patients with systemic sclerosis.

They found significantly higher HRV and lower circadian and spectral indices of HRV in systemic sclerosis patients, compared to control subjects.

Similarly in our study, we demonstrated an autonomic imbalance suggesting increased sympathetic or reduced parasympathetic activity demonstrated by time-domain HRV indices in primary RP patients compared to controls. Among time-domain variables of HRV indices, the mean of all fiveminute standard deviations of N–N (normal R–R) intervals during the 24-hour period (SDNN index) and the proportion of adjacent normal R–R intervals < 50 ms (pNN50) were found to be most associated with primary RP.

## Conclusion

The current study demonstrated significant differences in time-domain parameters of HRV analysis during asymptomatic 24-hour intervals and indicated the presence of an autonomic imbalance (increased sympathetic and decreased parasympathetic activity) in patients with primary RP compared to healthy controls. The exact mechanism of the relationship between primary RP and autonomic imbalance remains unclear and needs further studies. Future prospective studies may be helpful to demonstrate the role of HRV analysis in evaluating the progression and treatment effectiveness of patients with primary RP.
